# IL-6 and IL-10 gene polymorphisms and cirrhosis of liver risk from a comprehensive analysis

**DOI:** 10.1186/s12902-021-00906-3

**Published:** 2021-12-09

**Authors:** Minghui Zheng, Weizhen Fang, Menglei Yu, Rui Ding, Hua Zeng, Yan Huang, Yuanyang Mi, Chaohui Duan

**Affiliations:** 1 Department of Clinical Laboratory, Sun Yat-sen Memorial Hospital of Sun Yat-sen University, Guangzhou, 510120 China; 2 Guangdong Provincial Key Laboratory of Malignant Tumor Epigenetics and Gene Regulation, Sun Yat-sen Memorial Hospital of Sun Yat-sen University, Guangzhou, 510120 China; 3 Emergency Department, Sun Yat-Sen Memorial Hospital, Sun Yat-Sen University, Guangzhou, People’s Republic of China; 4 Department of Parasitology, Zhongshan School of Medicine, Sun Yat-sen University, Guangzhou, Guangdong 510080 People’s Republic of China; 5 Key Laboratory of Tropical Disease Control (Sun Yat-sen University), Chinese Ministry of Education, Guangzhou, Guangdong 510080 People’s Republic of China; 6 Department of Urology, Affiliated Hospital of Jiangnan University, 1000 Hefeng Rd, Wuxi, 214000 People’s Republic of China

**Keywords:** Interleukin-10, Interleukin-6, Cirrhosis of liver, Polymorphism, meta-analysis, Risk

## Abstract

**Background:**

Different inflammatory and immune cytokines play a key role in the development of cirrhosis of liver (CL). To investigate the association between interleukin-6,10 (IL-6,10) genes polymorphisms and CL risk through comparison of the allele and genotype distribution frequencies by meta-analysis.

**Methods:**

A literature search covered with the PubMed, Embase, Cochrane Library, Web of Science, Google Scholar, SinoMed (CNKI and Wanfang) through 20th April, 2021. Odds ratios (OR) and 95% confidence intervals (CI) were used to assess the strength of associations.

**Results:**

After a comprehensive search, three common polymorphisms (rs1800872, rs1800871, rs1800896) in IL-10 gene were selected, and three common polymorphisms (rs1800795, rs1800796, rs1800797) in IL-6 gene were also identified. The important finding was that IL-10 rs1800872 was a risk factor for CL development. For example, there has a significantly increased relationship between rs1800872 polymorphism and CL both in the whole group (OR: 1.30, 95%CI: 1.01–1.67 in heterozygote model), Asian population (OR: 1.40, 95%CI: 1.03–1.88 in heterozygote model) and hospital-based source of control (OR: 1.40, 95%CI: 1.01–1.96 in dominant model). In addition, significant association was found between rs1800896 and primary biliary cirrhosis subtype disease (OR: 1.30, 95%CI: 1.01–1.68 in allelic contrast model). No association was observed in all three polymorphisms in IL-6 gene.

**Conclusion:**

Our present study suggests that the IL-10 rs1800872 and rs1800896 polymorphisms is potentially associated with the risk of CL susceptibility.

## Background

Cirrhosis is characterized by extreme liver scarring (fibrosis), loss of organ function and serious complications related to portal hypertension (high blood pressure in the hepatic portal vein and its branches) [1].

Cirrhosis is the 11th leading cause of death worldwide, with a total burden of about 123 million deaths, of which about one tenth is decompensated [2, 3]. Liver cirrhosis (LC) is a severe public health problem worldwide, which is correlated with higher morbidity and mortality [4, 5]. The most common causes were chronic viral hepatitis [including infectious Hepatitis B virus (HBV, 39.64 million), and infectious Hepatitis C virus (HCV, 30.36 million)], alcoholic liver disease (26.04 million) and nonalcoholic fatty liver disease (NAFLD, 10.26 million), and other causes (16.62 million) [6]. With the implementation of HBV vaccination program and the application of effective anti HBV and HCV drugs in high endemic areas, the rate of liver cirrhosis caused by hepatitis gradually decreased, and the number of cases caused by NAFLD gradually increased [7]. NAFLD is now the commonest etiology worldwide, affecting 1 in 4 adults [8], and the progressive form that leads to patient with NAFLD, is predicted to increase by 63% between 2015 and 2030, representing a global cohort of at least 100 million individuals [9].

In the absence of effective intervention, cirrhosis can progress to decompensation, with ascites, gastrointestinal bleeding, hepatic encephalopathy, hepatorenal syndrome, and liver cancer [7]. Liver transplantation is the most effective therapeutic option for end-stage liver disease but is a scarce resource [1]. Moreover, although antifibrotic or pro-regenerative drug therapies for cirrhosis have been approved, they have been in clinical trials and the effect has not been determined [1].

Cytokines, such as interleukins, play an integral role in the host immune response and may be a critical factor in determining the duration and severity of virus infection, fibrosis formation [10, 11]. Interleukin-10 (IL-10) is an important anti-inflammatory cytokine secreted by different cells such as liver cells, T regulatory lymphocytes, activated macrophages, and T helper (Th) 2 cells [12]. It inhibits macrophage-dependent antigen presentation, proliferation of T-lymphocytes, and Th1 cytokine secretion and acts as an inhibitor of Th1 effectors mechanism [12]. Three common polymorphisms -1082G/A (rs1800896), − 819C/T (rs1800871), and − 592 C/A (rs1800872) related to cirrhosis of liver (CL) have been wildly reported [13]. IL-6, a primary immunomodulatory cytokine, has been documented to play a pivotal role in regulating the biological processes of many cells including hepatocytes [14]. Three common polymorphisms -174G/C (rs1800795), − 572G/C (rs1800796), and − 597 G/A (rs1800797) related to CL have been wildly reported [15].

In order to overcome the factors of sample size, regional and ethnic differences, our study summarized all published literatures related to the relationship between IL-6 and IL-10genes polymorphisms and CL by meta-analysis, to comprehensively evaluate the relationship between several polymorphisms and CL, and to provide evidence-based medical research basis for the etiology of CL.

## Materials and methods

### Literature search strategy

A computerized literature search was performed for relevant studies from PubMed, Embase, Cochrane Library, Web of Science, Google Scholar, SinoMed (CNKI and Wanfang) before 20th April, 2021. The following keywords were jointly used “interleukin 10 or interleukin 6 or IL-10 or IL-6”, “polymorphism or variation or mutation”, “rs1800795 or rs1800796 or rs1800797 or rs1800896 or rs1800871 or rs1800872” and “live cirrhosis or primary biliary cirrhosis or nonalcoholic fatty liver disease”. If studies applied the same case clinic information, only the largest sample size was selected [16].

### Inclusion criteria

The included studies met the following criteria: (a) there were clear criteria for the diagnosis of CL, such as B-ultrasound, CT, MRI, endoscopic retrograde cholangiopancreatography, liver biopsy, and so on, (b) the correlation between CL risk and IL-10 and IL-6 genes polymorphisms (rs1800795 or rs1800796 or rs1800797 or rs1800896 or rs1800871 or rs1800872), (c) case-control or cohort design, (d) provided sufficient data for calculating odds ratio (OR) with 95% confidence interval (95%CI), and (e) duplicated studies with the same cases [17].

### Data extraction

The following information was extracted from each included study: name of the first author, publication year, country of origin, ethnicity, numbers of cases and controls, HWE of control group, genotype method and sub-type of CL. The data were selected independently by 2 investigators who reached a consensus on all items [18].

### Statistical analysis

The associations of the IL-10 and IL-6 genes polymorphisms and risk of CL were estimated by calculating the OR and 95%CI. The statistical significance of the OR was determined with the *Z* test [19]. The significance of the effect for correlation was determined by the *Z* test [18]. The heterogeneity among studies was evaluated using a Q test and *I*^2^ test as described in other studies [20, 21]. As a guide, *I*^2^ values of <25% may be considered ‘low’, value of ~ 50% may be considered ‘moderate’ and values of >75% may be considered ‘high’ [22]. The Mantel-Haenszel (fixed effect) model was chosen, otherwise, if *P*_heterogeneity_ < 0.1, the random effects (DerSimonian-Laird) model was applied [23, 24]. Sensitivity analysis was undertaken by removing each study once to assess whether any single study could influence the stability of results [25]. The departure of frequencies of six polymorphisms from expectation under HWE was assessed by the Pearson’s χ^2^ test, *P* < 0.05 was considered significant [26]. Begg’s funnel plots and Egger’s regression test were performed to estimate the potential publication bias [27]. All statistical tests for this meta-analysis were performed using version 10.0 Stata software (StataCorp LP, College Station, TX, USA) [18].

### Network of protein-interaction of IL-6 and IL-10 gene

To more complete understanding of the role of IL-6 and IL-10 in CL, the network of gene-gene interactions for IL-6 and IL-10 was predicted through online String database (http://string-db.org/) [28].

## Results

### Study searching and their basic information

**Fig. 1:**
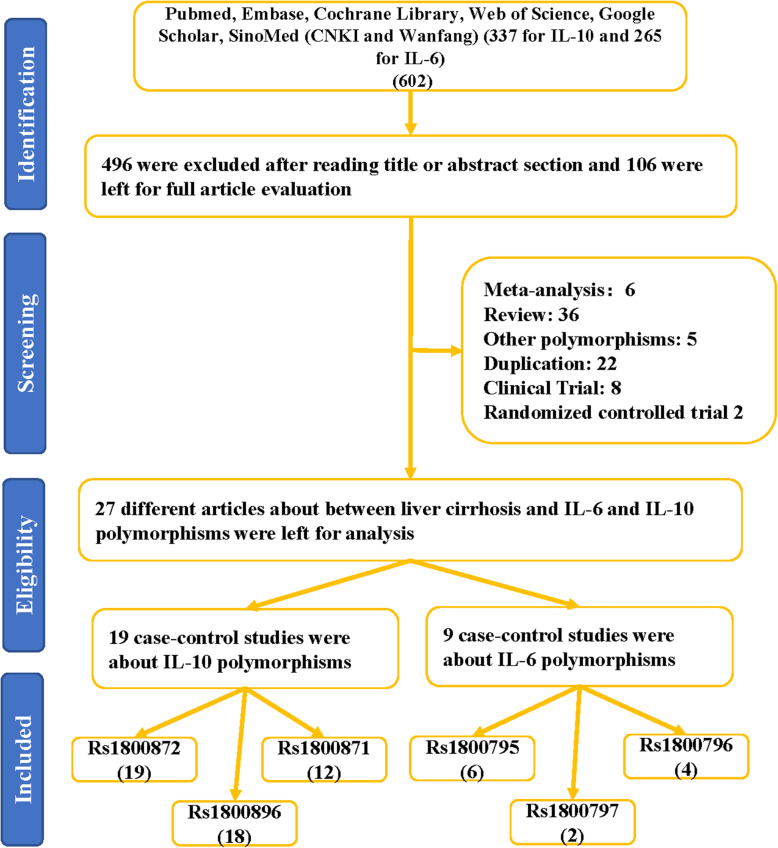
Flowchart illustrating the search strategy used to identify association studies for IL-10 and IL-6 polymorphisms and CL risk

**Fig. 2:**
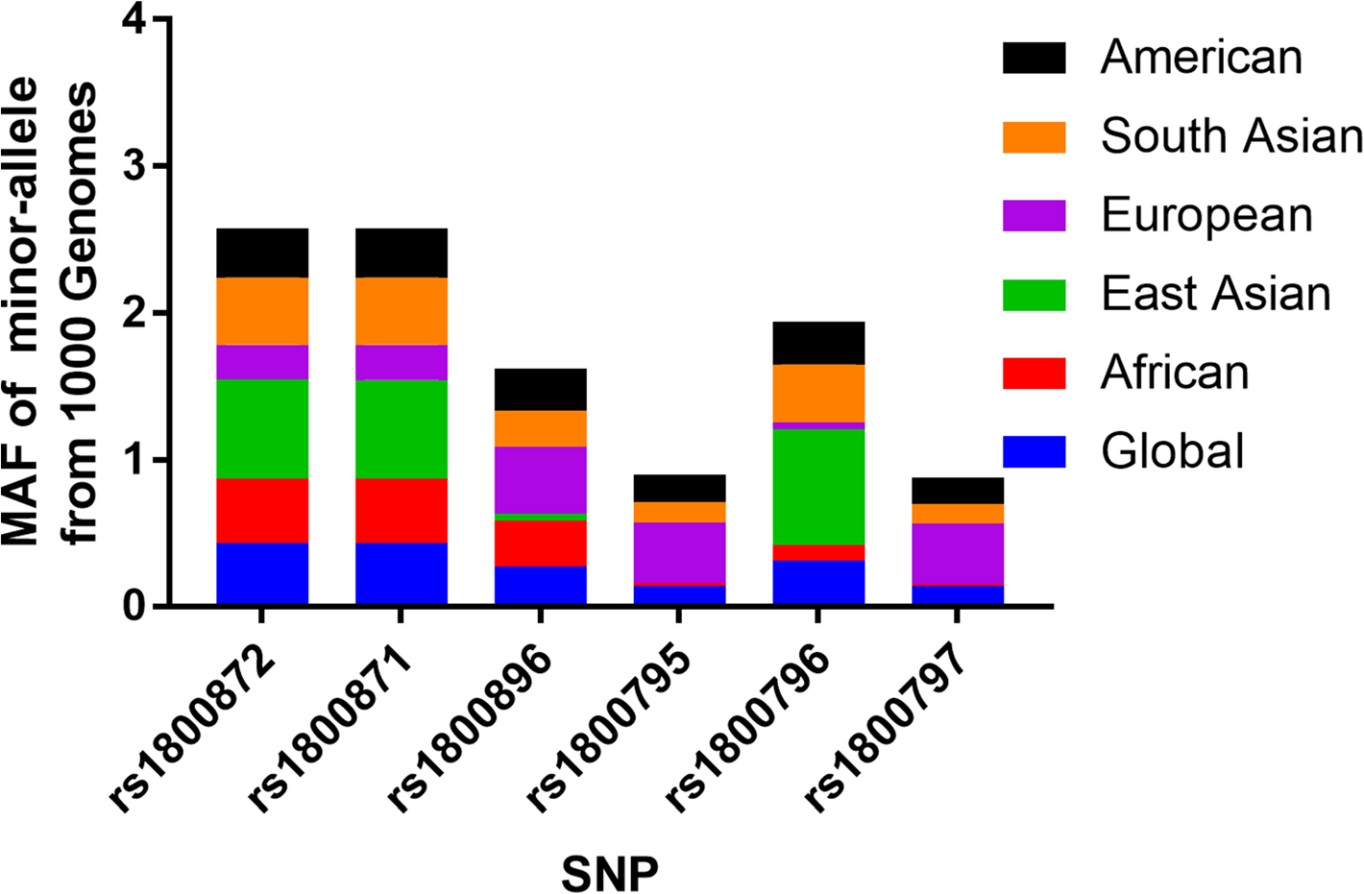
The MAF of minor-allele (mutant-allele) for IL-10 and IL-6 polymorphisms from the 1000 Genomes online database and present analysis

As depicted in Fig. 1, 602 articles were garnered by PubMed, Embase, Cochrane Library, Web of Science, Google Scholar, SinoMed (CNKI and Wanfang (337 titles about IL-10 gene polymorphisms and 265 titles for IL-6 gene polymorphisms) database. 496 obviously irrelevant articles were excluded after screening the titles and abstract sections. The full texts were then evaluated, and 79 additional articles were further excluded as they were duplication (22), meta-analysis systematic analysis or review (42), other polymorphisms (5), clinical trial (8) and randomized controlled trial (2). Finally, 27 different articles [15, 29–55] met the inclusion criteria and were included in our meta-analysis. Among these included studies, 19 studies were performed about IL-10 three polymorphisms (19 case-control studies for rs1800872, 12 for rs1800871, 18 for rs1800896), and 9 studies was related to IL-6 three polymorphisms (6 for rs1800795, 4 for 1,800,796 and 2 for rs1800797). All the included studies used blood samples for DNA extraction (Table 1). In addition, we checked the Minor Allele Frequency (MAF) reported for the six main worldwide populations in the 1000 Genomes Browser (https://www.ncbi.nlm.nih.gov/snp/) (Fig. 2). The genotyping methods included polymerase chain reaction-restrictive fragment length polymorphism, sequencing, TaqMan, Sequenom Assay Design, amplification refractory mutation system and sequence specific primer.

**Table 1:** Characteristics of included studies about polymorphisms in IL-6 and IL-10 genes and cirrhosis of liver risk

Author	Year	Country	Ethnicity	Case	Control	SOC	HWE	Genotype	Sub-type
-592	rs1800872								
Chen	2004	China	Asian	77	54	HB	0.633	PCR-RFLP	PBC
Zappala	1998	UK	Caucasian	171	141	HB	0.071	PCR	PBC
Matsushita	2002	USA	Caucasian	94	72	PB	0.501	PCR-RFLP	PBC
Matsushita	2002	USA	Caucasian	65	71	PB	< 0.01	PCR-RFLP	PBC
Marcos	2008	Spain	Caucasian	96	100	HB	0.093	PCR-RFLP	ALC
Yao	2015	China	Asian	318	318	PB	< 0.01	PCR-RFLP	LC
Barooah	2020	India	Asian	96	110	HB	0.009	PCR-RFLP	HCV-LC
Liu	2015	China	Asian	192	192	HB	< 0.01	Sequenom Assay Design	Mixed
Cao	2016	China	Asian	241	254	HB	< 0.01	PCR-RFLP	LC
Baghi	2015	Iran	Asian	9	102	PB	0.664	PCR-RFLP	HBV-LC
Cheong	2005	South Korea	Asian	79	261	HB	< 0.01	PCR	HBV-LC
Sheneef	2017	Egypt	African	50	50	PB	0.889	ARMS-PCR	HCV-LC
Corchado	2013	Korea	Asian	39	49	HB	0.187	PCR	HCV-LC
Fan	2004	China	Asian	77	160	HB	< 0.01	PCR-RFLP	PBC
Khalifa	2016	Saudi Arabia	Asian	109	110	HB	0.525	PCR-RFLP	HBV-LC
Moreira	2016	Brazil	Mixed	37	102	HB	0.316	PCR-SSP	HCV-LC
Wang	2010	China	Asian	50	42	HB	< 0.01	PCR	HBV-LC
Jiang	2009	China	Asian	169	119	HB	0.552	PCR-RFLP	HBV-LC
Wu	2010	China	Asian	50	96	HB	0.125	PCR-RFLP	HBV-LC
−819	rs1800871								
Chen	2004	China	Asian	77	54	HB	1	PCR-RFLP	PBC
Matsushita	2002	USA	Caucasian	94	72	PB	0.501	PCR-RFLP	PBC
Matsushita	2002	USA	Caucasian	65	71	PB	0.049	PCR-RFLP	PBC
Yao	2015	China	Asian	318	318	PB	0.227	PCR-RFLP	LC
Barooah	2020	India	Asian	96	110	HB	0.474	PCR-RFLP	HCV-LC
Liu	2015	China	Asian	192	192	HB	0.073	Sequenom Assay Design	Mixed
Baghi	2015	Iran	Asian	9	102	PB	0.369	PCR-RFLP	HBV-LC
Cheong	2005	South Korea	Asian	79	261	HB	0.458	PCR	HBV-LC
Yang	2013	China	Asian	40	64	PB	0.821	ARMS-PCR	ALC
Fan	2004	China	Asian	77	160	HB	0.455	PCR-RFLP	PBC
Moreira	2016	Brazil	Mixed	37	102	HB	0.316	PCR-SSP	HCV-LC
Wang	2010	China	Asian	50	43	HB	0.017	PCR	HBV-LC
−1082	rs1800896								
Chen	2004	China	Asian	77	54	HB	0.611	PCR-RFLP	PBC
Bathgate	2000	UK	Caucasian	61	330	HB	0.003	sequence	PBC
Matsushita	2002	USA	Caucasian	94	72	PB	0.859	PCR-RFLP	PBC
Matsushita	2002	USA	Caucasian	65	71	PB	0.568	PCR-RFLP	PBC
Abd El-Baky	2020	Egypt	African	22	48	PB	< 0.01	TaqMan real-time PCR	HCV-LC
Yao	2015	China	Asian	318	318	PB	0.898	PCR-RFLP	LC
Barooah	2020	India	Asian	96	110	HB	0.054	PCR-RFLP	HCV-LC
Liu	2015	China	Asian	266	532	HB	< 0.01	Sequenom Assay Design	Mixed
Cao	2016	China	Asian	241	254	PB	0.953	PCR-RFLP	LC
Baghi	2015	Iran	Asian	9	102	PB	0.047	PCR-RFLP	HBV-LC
Cheong	2005	South Korea	Asian	79	261	HB	0.769	PCR	HBV-LC
Yang	2013	China	Asian	40	64	PB	0.452	ARMS-PCR	ALC
Sheneef	2017	Egypt	African	50	50	PB	0.259	ARMS-PCR	HCV-LC
Fan	2004	China	Asian	77	160	HB	0.505	PCR-RFLP	PBC
Khalifa	2016	Saudi Arabia	Asian	109	110	HB	0.267	PCR-RFLP	HBV-LC
Moreira	2016	Brazil	Mixed	37	102	HB	0.973	PCR-SSP	HCV-LC
Wang	2010	China	Asian	50	42	HB	0.874	PCR	HBV-LC
Wu	2010	China	Asian	50	96	HB	0.629	PCR-RFLP	HBV-LC
-174G > C									
Giannitrapani	2011	Italy	Caucasian	95	105	HB	0.776	PCR-RFLP	LC
Fan	2004	China	Asian	77	160	PB	< 0.01	SSP	PBC
Falleti	2008	Italy	Caucasian	219	236	PB	0.536	PCR-RFLP	Mixed
Marcos	2009	Spain	Caucasian	96	160	PB	0.333	TaqMan	ALC
Motawi	2016	Egypt	African	65	100	HB	< 0.01	PCR-RFLP	HCV-LC
Moreira	2016	Brazil	Mixed	38	100	HB	0.718	PCR-SSP	HCV-LC
IL6–572									
Park	2003	Korea	Asian	696	280	PB	0.193	sequence	HBV-LC
Falleti	2008	Italy	Caucasian	219	236	PB	0.249	PCR-RFLP	Mixed
Saxenas	2014	India	Asian	63	83	HB	< 0.01	PCR-RFLP	HBV-LC
Tang	2013	China	Asian	153	265	HB	0.529	TaqMan	HBV-LC
597G > A									
Falleti	2008	Italy	Caucasian	219	236	PB	0.348	PCR-RFLP	Mixed
Saxenas	2014	India	Asian	3	138	HB	0.613	PCR-RFLP	HBV-LC

HB: hospital-based; PB: population-based; SOC; source of control; PCR-RFLP: polymerase chain reaction followed by restriction fragment length polymorphism; SSP: sequence specific primer; ARMS: amplification refractory mutation system; HWE: Hardy-Weinberg equilibrium of control group; PBC: primary biliary cirrhosis; LC: liver cirrhosis; ALC: alcoholic liver cirrhosis, HCV: hepatitis C virus infection, HBV: hepatitis B virus infection

### Quantitative synthesis

#### IL-10 − 592 polymorphism

**Fig. 3:**
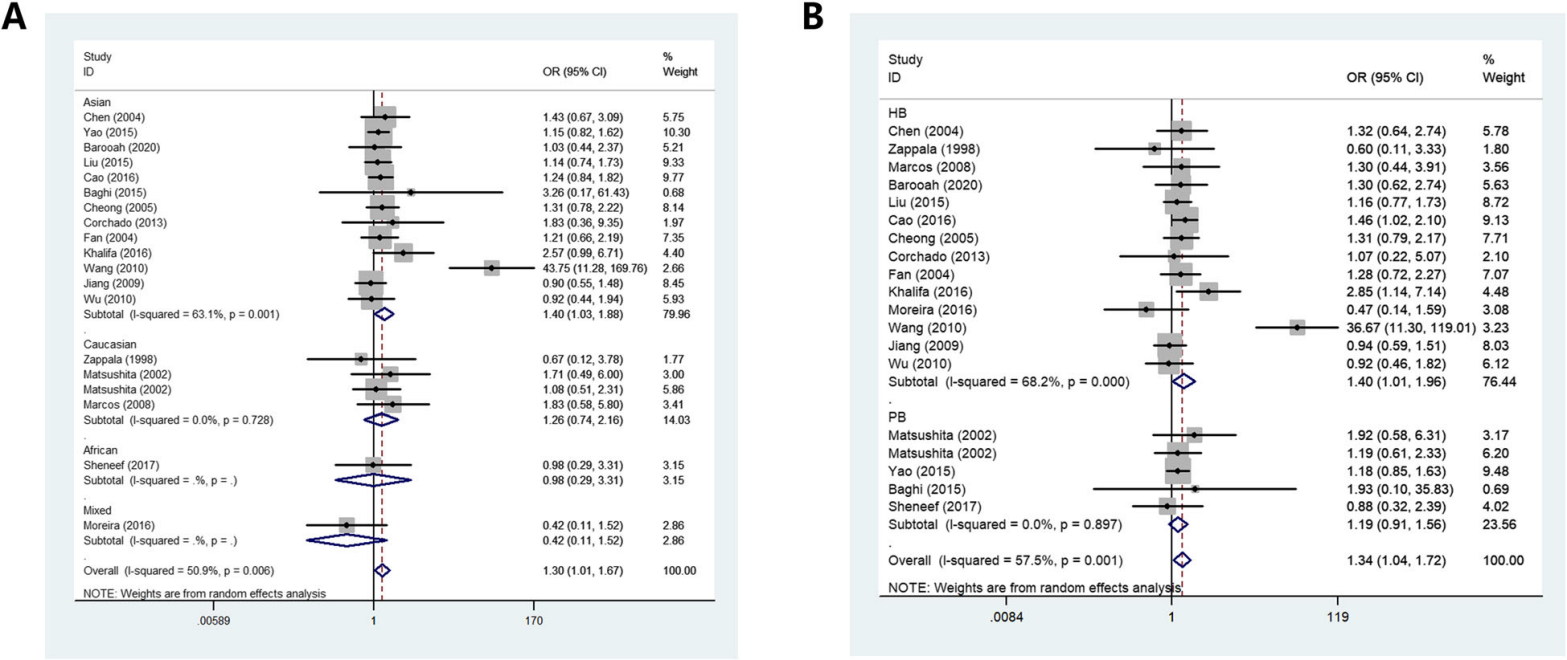
Forest plot of CL risk associated with IL-10 gene −592 polymorphism A: heterozygote comparison model in total analysis and in ethnicity subgroup; B: dominant model in source of control

In whole analysis, increased associations were observed in two genetic models (heterozygote comparison: OR: 1.30, 95%CI:1.01–1.67, *P* = 0.006 for heterogeneity, *P* = 0.039, *I*^2^ = 50.9%, Fig. 3A; dominant model: OR: 1.34, 95%CI:1.04–1.72, *P* = 0.001 for heterogeneity, *P* = 0.021, *I*^2^ = 57.5%). In subgroup analysis by ethnicity, based on different frequency of races, there also had increased associations between this polymorphism and CL in Asians not Caucasians in all models (allelic contrast: OR: 1.25, 95%CI:1.01–1.55, *P* = 0.000 for heterogeneity, *P* = 0.042, *I*^2^ = 72.3%; heterozygote comparison: OR: 1.40, 95%CI:1.03–1.88, *P* = 0.001 for heterogeneity, *P* = 0.029, *I*^2^ = 63.1%, Fig. 3A; dominant model: OR: 1.47, 95%CI:1.09–1.99, *P* = 0.000 for heterogeneity, *P* = 0.013, *I*^2^ = 68.3%). In addition, regular analysis by source of control, also significantly trend was found for this SNP in HB rather than PB studies (dominant model: OR: 1.40, 95%CI:1.01–1.96, *P* = 0.000 for heterogeneity, *P* = 0.046, *I*^2^ = 68.2%, Fig. 3B). Finally, many causes may result in cirrhosis, such as primary biliary cirrhosis (PBC), alcoholics with liver cirrhosis, HCV-LC, HBV-LC and immune cirrhosis, to our regret, no significant association was found in all kinds of this subgroup (Table 2).

**Table 2:** Stratified analyses of IL-6 and IL-10 genes’ common polymorphisms on cirrhosis of liver risk

Variables	N	Case/	Allelic contrast	Heterozygote comparison	Dominant model
		Control	OR(95%CI) *P*_h_ *P I*-squared	OR(95%CI) *P*_h_ *P I*-squared	OR(95%CI) *P*_h_ *P I*-squared
IL-10 -592					
Total	19	2019/2403	1.15 (0.98–1.37)0.000 0.093 65.7%	1.30 (1.01–1.67)0.006 0.03950.9%	1.34 (1.04–1.72)0.001 0.02157.5%
Ethnicity					
Asian	13	1506/1867	1.25 (1.01–1.55)0.000 0.042 72.3%	1.40 (1.03–1.88)0.001 0.02963.1%	1.47 (1.09–1.99)0.000 0.01368.3%
Caucasian	4	426/384	0.98 (0.78–1.22)0.270 0.840 23.4%	1.26 (0.74–2.14)0.728 0.399 0.0%	1.24 (0.76–2.02)0.747 0.395 0.0%
SOC					
HB	14	1483/1790	1.19 (0.95–1.48)0.000 0.125 73.6%	1.36 (0.98–1.89)0.001 0.06863.5%	1.40 (1.01–1.96)0.000 0.04668.2%
PB	5	536/613	1.11 (0.93–1.33)0.594 0.234 0.0%	1.17 (0.88–1.57)0.917 0.277 0.0%	1.19 (0.91–1.56)0.897 0.208 0.0%
Disease type					
PBC	5	484/498	1.11 (0.91–1.35)0.590 0.319 0.0%	1.23 (0.85–1.78)0.908 0.281 0.0%	1.27 (0.89–1.79)0.871 0.184 0.0%
HBV-LC	6	466/730	1.46 (0.86–2.49)0.000 0.163 35.9%	2.24 (0.95–5.28)0.000 0.06584.0%	2.26 (0.95–5.38)0.000 0.06586.3%
HCV-LC	4	222/311	0.98 (0.75–1.28)0.161 0.901 41.8%	0.93 (0.53–1.64)0.531 0.794 0.0%	0.98 (0.59–1.62)0.572 0.926 0.0%
-819					
Total	12	1134/1549	1.07 (0.88–1.30)0.017 0.485 52.4%	1.14 (0.86–1.51)0.072 0.35440.3%	1.15 (0.86–1.53)0.029 0.35448.8%
Ethnicity					
Asian	9	938/1304	1.05 (0.82–1.34)0.006 0.089 63.0%	1.18 (0.86–1.64)0.047 0.30449.0%	1.17 (0.83–1.64)0.016 0.36757.2%
Caucasian	2	159/143	1.28 (0.90–1.83)0.790 0.173 0.0%	1.23 (0.64–2.34)0.542 0.537 0.0%	1.33 (0.74–2.39)0.493 0.334 0.0%
SOC					
HB	7	608/922	1.14 (0.88–1.47)0.037 0.321 55.2%	1.22 (0.78–1.91)0.020 0.38660.2%	1.23 (0.80–1.91)0.016 0.34361.5%
PB	5	526/627	0.96 (0.68–1.36)0.045 0.832 58.9%	1.07 (0.81–1.42)0.543 0.614 0.0%	1.08 (0.83–1.40)0.229 0.556 28.8%
Disease type					
PBC	4	313/357	1.24 (0.97–1.57)0.964 0.082 0.0%	1.37 (0.95–1.99)0.906 0.095 0.0%	1.38 (0.97–1.96)0.911 0.071 0.0%
HBV-LC	3	138/406	1.55 (0.55–4.43)0.004 0.409 81.6%	2.96 (0.70–12.47)0.0320.14070.8%	2.68 (0.67–10.74)0.0240.16573.2%
HCV-LC	2	133/212	0.92 (0.66–1.27)0.901 0.595 0.0%	0.65 (0.38–1.14)0.457 0.132 0.0%	0.71 (0.42–1.20)0.467 0.203 0.0%
-1082					
Total	18	1741/2776	1.01 (0.85–1.20)0.013 0.892 47.5%	1.01 (0.82–1.23)0.202 0.941 21.2%	1.00 (0.80–1.24)0.053 0.971 37.9%
Ethnicity					
Asian	12	1412/2103	0.94 (0.76–1.17)0.018 0.577 51.9%	1.01 (0.78–1.33)0.092 0.921 37.5%	0.96 (0.72–1.29)0.024 0.795 50.2%
Caucasian	3	220/473	1.25 (0.94–1.65)0.323 0.122 11.4%	1.20 (0.78–1.85)0.900 0.409 0.0%	1.30 (0.86–1.95)0.699 0.213 0.0%
African	2	72/98	1.27 (0.82–1.97)0.817 0.282 0.0%	1.12 (0.47–2.70)0.241 0.799 27.2%	1.24 (0.55–2.82)0.269 0.602 18.0%
SOC					
HB	10	902/1797	1.04 (0.89–1.21)0.502 0.601 0.0%	1.11 (0.90–1.37)0.734 0.319 0.0%	1.09 (0.90–1.32)0.683 0.380 0.0%
PB	8	839/979	0.99 (0.72–1.36)0.005 0.966 65.8%	0.86 (0.56–1.33)0.087 0.505 43.7%	0.87 (0.53–1.42)0.016 0.577 59.3%
Disease type					
PBC	5	374/687	1.30 (1.01–1.68)0.568 0.043 0.0%	1.32 (0.93–1.89)0.901 0.122 0.0%	1.39 (0.98–1.95)0.863 0.061 0.0%
HBV-LC	5	297/611	0.97 (0.71–1.32)0.318 0.827 15.2%	1.30 (0.89–1.90)0.527 0.170 0.0%	1.15 (0.80–1.67)0.420 0.447 0.0%
HCV-LC	4	205/310	0.98 (0.76–1.28)0.547 0.897 0.0%	0.81 (0.53–1.24)0.865 0.332 0.0%	0.85 (0.58–1.26)0.489 0.414 0.0%
LC	2	559/572	0.72 (0.60–0.85)0.987 0.000 0.0%	0.64 (0.44–0.93)0.865 0.019 0.0%	0.56 (0.39–0.80)0.892 0.001 0.0%
IL-6 -174					
Total	6	590/861	1.17 (0.73–1.86)0.002 0.511 77.5%	1.42 (0.70–2.87)0.000 0.330 78.3%	1.37 (0.71–2.63)0.001 0.346 77.2%
Ethnicity					
Caucasian	3	410/501	0.89 (0.73–1.09)0.631 0.244 0.0%	0.87 (0.65–1.15)0.314 0.316 13.7%	0.86 (0.66–1.12)0.550 0.257 0.0%
SOC					
HB	3	198/305	1.98 (0.55–7.05)0.001 0.294 86.8%	2.79 (0.41–18.88)0.000 0.294 90.4%	2.71 (0.47–15.57)0.0000.26589.6%
PB	3	392/556	0.99 (0.63–1.55)0.083 0.961 59.8%	1.04 (0.76–1.42)0.130 0.800 50.9%	0.98 (0.73–1.32)0.110 0.916 54.7%
−572					
Total	4	1131/864	1.15 (0.97–1.36)0.859 0.117 0.0%	2.23 (0.80–6.21)0.000 0.127 89.2%	1.60 (0.83–3.06)0.005 0.157 76.3%
−597					
Total	2	280/374	0.84 (0.66–1.08)0.453 0.168 0.0%	0.88 (0.63–1.23)0.203 0.462 38.3%	0.84 (0.61–1.15)0.278 0.283 15.0%

*P*_h_: value of *Q*-test for heterogeneity test; *P*: *Z*-test for the statistical significance of the OR

#### IL-10 -1082 polymorphism

**Fig. 4:**
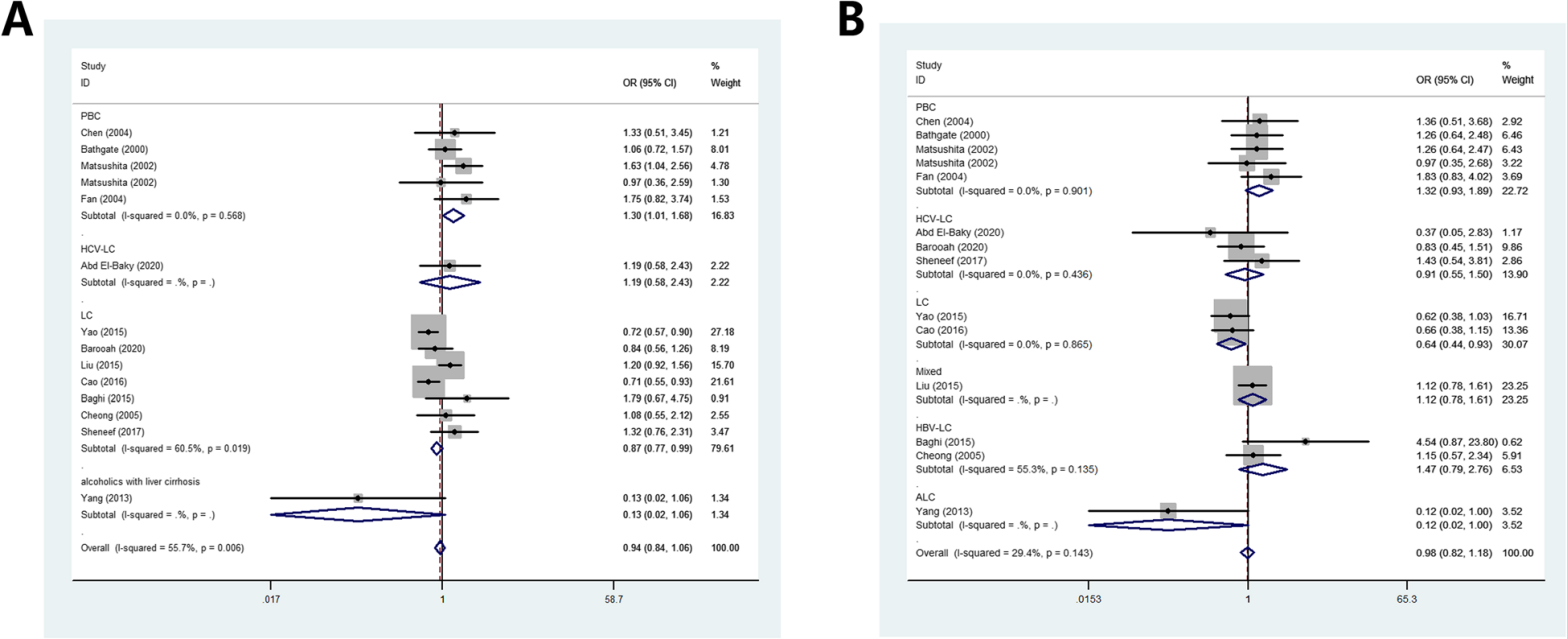
Forest plot of CL risk associated with IL-10 gene − 1082 polymorphism from allelic contrast in sub-type analysis. A: PBC in the allelic contrast model; B: LC in the allelic contrast model

No association was detected in total, ethnicity, source of control subgroups, however, in the subgroup of disease type subgroup, increased relationship was observed in the allelic contrast model (OR: 1.30, 95%CI:1.01–1.68, *P* = 0.568 for heterogeneity, *P* = 0.043, *I*^2^ = 0.0%) (Fig. 4A). In the sub-type of CL, we found decreased association was existed in LC risk and this polymorphism (such as OR: 0.64, 95%CI:0.44–0.93, *P* = 0.865 for heterogeneity, *P* = 0.019, *I*^2^ = 0.0%, Fig. 4B).

#### IL-10-819, IL-6 -174, − 572 and − 597 polymorphisms

No association was found in above four kinds of polymorphisms (data not shown) (Table 2).

### Bias diagnosis for publication and sensitivity analysis

**Fig. 5:**
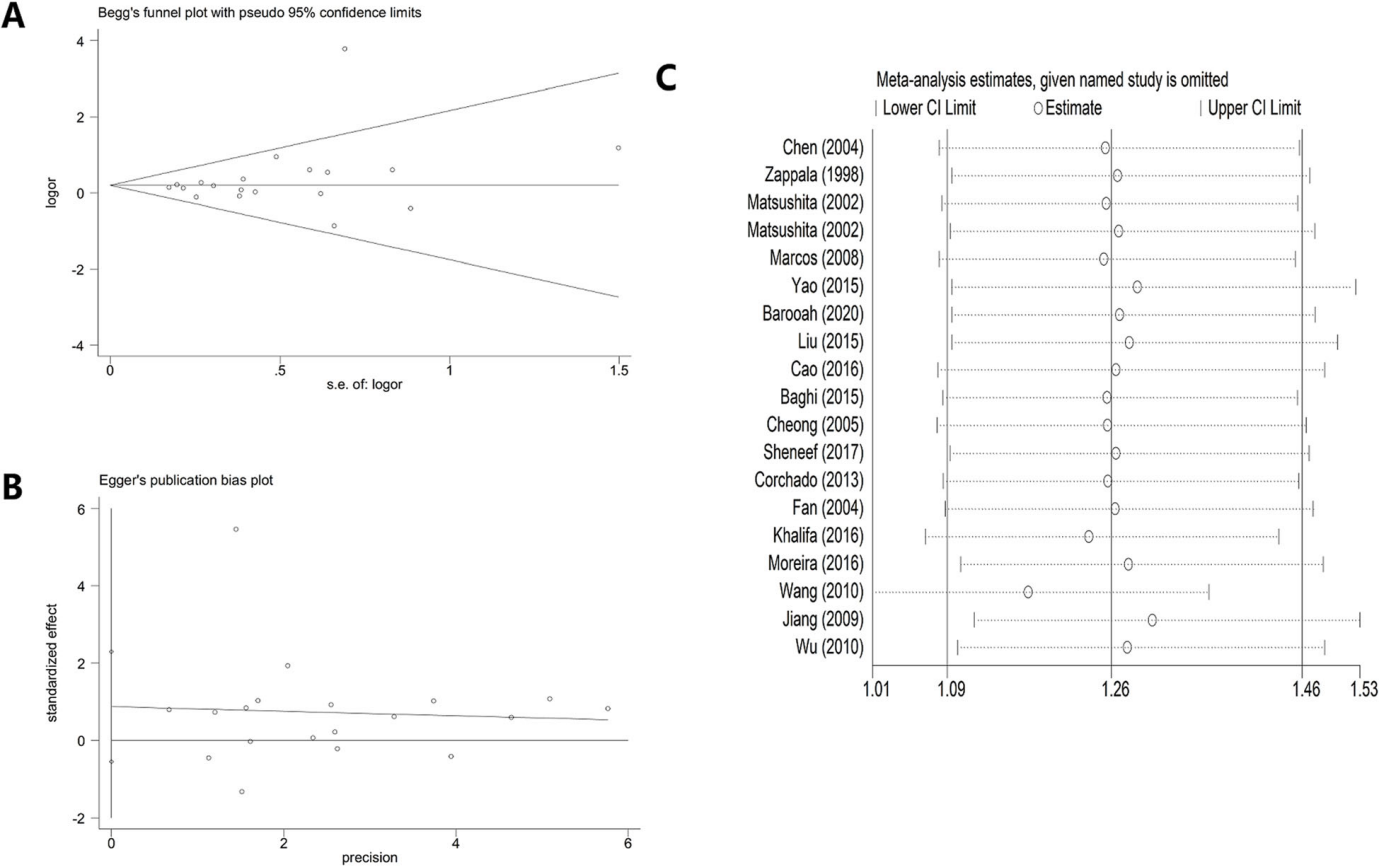
A: Begg’s funnel plot for publication bias test. Each point represents a separate study for the indicated association. Log [OR], natural logarithm of OR. Horizontal line, mean effect size. B: Egger’s publication bias plot. C: Sensitivity analysis between IL-10 gene − 592 polymorphism and CL risk

The publication bias was evaluated by both Begg’s funnel plot and Egger’s test (such as − 592 polymorphism). At beginning, the shape of the funnel plots seemed asymmetrical in allele comparison for − 592 by Begg’s test, suggesting no publication bias was existed. Then, Egger’s test was applied to provide statistical evidence of funnel plot symmetry. As a result, no obvious evidence of publication bias was observed (such as allelic contrast: t = 2.57, *P* = 0.024 for Egger’s test; *z* = 1.75, *P* = 0.08 for Begg’s test (Fig. 5 A, B) (Table 3).

**Table 3:** Publication bias tests (Begg’s funnel plot and Egger’s test for publication bias test) for IL-10 -592 polymorphism

Egger’s test						Begg’s test	
Genetic type	Coefficient	Standard error	t	*P* value	95%CI of intercept	z	*P* value
C-allele vs. A-allele	−0.181	1.211	−0.15	0.883	(−2.736–2.374)	1.26	0.208
CA vs. AA	−0.047	0.447	−0.11	0.917	(−0.992–0.897)	0.35	0.726
CC + CA vs. AA	−0.047	0.51	−0.09	0.927	(−1.124–1.029)	0.56	0.576

To delete studies which may influence the power and stability of whole study, we applied the sensitive analysis (such as − 592 polymorphism), finally, no sensitive case-control studies were found for − 592 SNP in three models (Fig. 5C).

### Gene-gene network diagram and interaction of online website

**Fig. 6:**
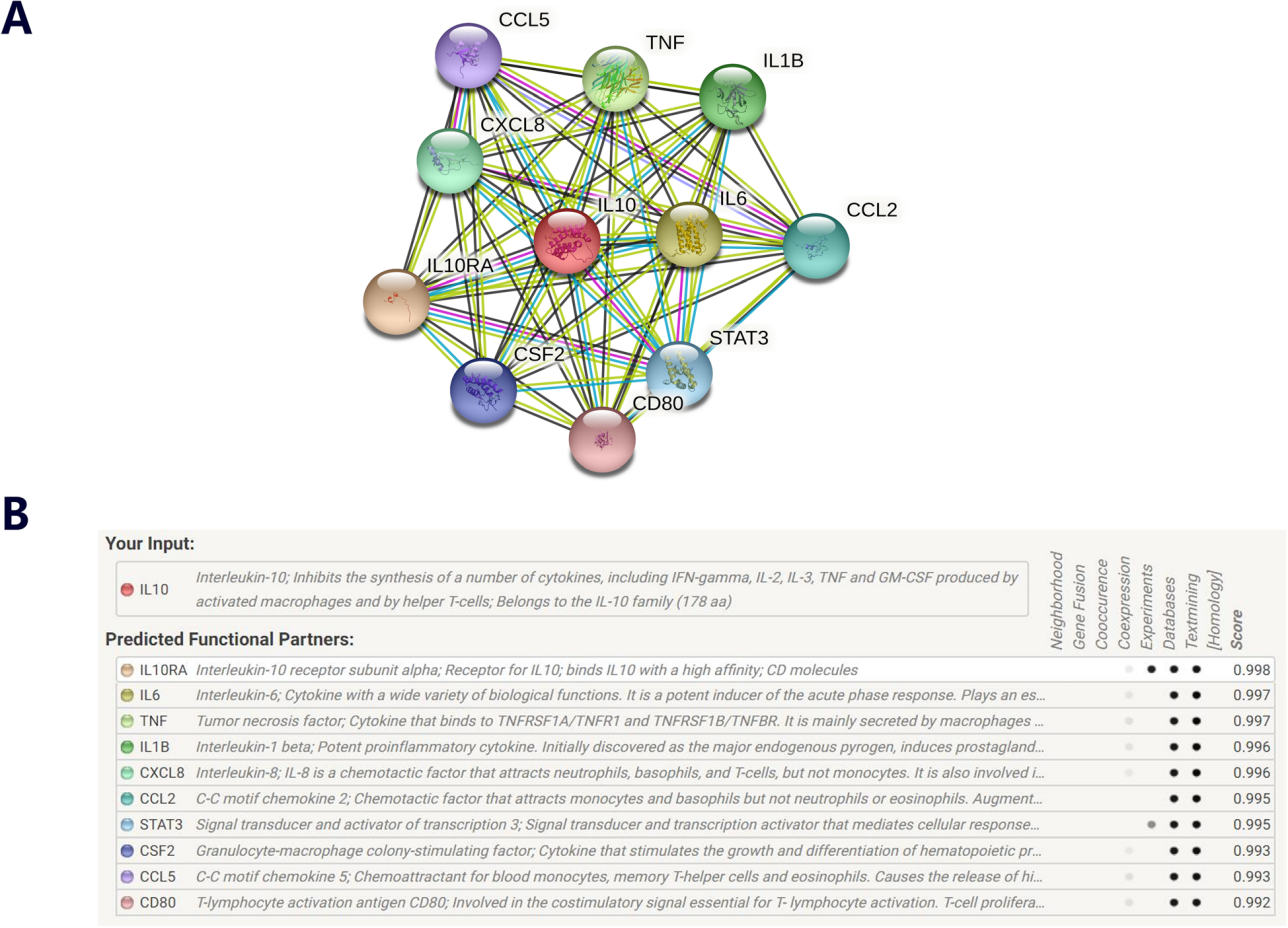
Human IL-10 and IL-6 interactions network with other genes obtained from String server. At least 9 genes have been indicated to correlate with. IL10RA: interleukin-10 receptor subunit aloha; TNF: tumor necrosis factor; IL1B: interleukin-1 beta; CXCL8: interleukin-8; CCL2: C-C motif chemokine 2; STAT3: sugnal transducer and activator of transcription 3; CSF2: granulocyte-macrophage colony-stimulating factor; CCL5: C-C motif chemokine 5; CD80: T-lymphocyte activation antigen CD80

String online server indicated that IL-10 and IL-6 gene interacts with numerous genes. The network of gene-gene interaction has been illustrated in Fig. 6.

## Discussion

Cirrhosis is the final stage of liver fibrosis, which itself results from a perpetuated wound-healing process after a liver injury that can lead to a wide range of chronic diseases involving the liver [56, 57]. In addition, cirrhosis is a burden on the individual and on public health. To our knowledge, the most prevalent chronic liver diseases are chronic viral hepatitis (from hepatitis B or C infection), alcohol-related liver disease, and NAFLD [56].

Cirrhosis negatively impacts on patient reported outcomes and health-related quality of life [58–60]. The impact of cirrhosis on quality of life can add to the existing impairment of quality of life related to viraemia in patients with hepatitis C [61, 62]. Conversely, effective treatment of hepatitis C can lead to significant gains in patients’ quality of life, especially for patients with decompensated cirrhosis. In addition, there is evolving evidence indicating that quality of life is significantly impaired in patients with NAFLD in the form of non-alcoholic steatohepatitis [63]. Nowadays, the trends indicate that the contribution from NAFLD related cirrhosis is increasing within cirrhosis. Other risk factors, such as substantial regional variation, and substantial variation in time trends in the prevalence of these etiology, should also been paid attention.

We devoted to find some susceptible factors, finally, we focused on two cytokines (IL-6 and IL-10). So far, multiple genes have been shown to be associated with increased liver disease risk, such as CTLA-4, IL-18, transmembrane 6 superfamily member 2 and GSTM1 [64–66]. Besides, more and more studies have indicated IL-6 and IL-10 polymorphisms may be associated with CL risk. Due to the limited number of samples about each study, the conclusion for every study may not be credible. Yao et al. found that IL-10 rs1800896 polymorphism was correlated with an increased risk of CL, especially in individuals with chronic hepatitis B [46]. Falleti et al. polymorphisms of IL-6 were associated with hepatocellular carcinoma (HCC) occurrence among patients with CL [34]. It is necessary to combine all previous studies and increase the sample size, we wish to obtain comprehensive and convince conclusions between IL-6 or IL-10 polymorphism and CL susceptibility.

It is in time to analyze the association between IL-6 and IL-10 polymorphisms and CL risk using meta-analysis method. After our searching through main database, 19 different case-control studies were identified for IL-10 polymorphism, and 9 case-control studies were detected for IL-6 polymorphism. The main results about current study are that IL-10 -592 polymorphism was a risk factor for CL risk in the whole samples, especially in Asian population, moreover, IL-10 − 1082 polymorphism had an increased association for PBC, which may offer references for early detection, prevention and treatment about CL. No positive results were observed in other polymorphisms, which due to the sample size and publication bias.

We all know the development and outcome about CL is complex and multi-factorial. Focusing on only each gene or each polymorphism is limited. Hence, we try our best to detect other potential genes related with CL based on online String server. Other nine most possible genes and current two related genes were shown in the network. Among them, six genes belong to cytokine family, and these scores were all in the front, the first related genes are IL-10RA, which is the receptor of IL-10 gene. Hennig et al. indicated IL-10RA gene polymorphisms may play a modulatory role in the outcome (including severity of fibrosis and overall inflammation) of hepatitis C infection [67]. Galal et al. confirmed that TNF family lymphotoxin-alpha GG genotype and low platelet count were independent predictors for HCC development in patients with HCV-LC [68]. Amirpour-Rostami et al. summarized the main correlation between the polymorphisms within IL-18 and IL-1B genes and chronic hepatitis B [69]. In a word, we should deep explore these partners of IL-10 and 6 genes, and gene-gene interactions in the development and treatment for CL in the next step.

There are some limitations should be paid attention. At the beginning, further studies should focus on Mixed and African populations, which was vacant in current analysis and need many more studies. Second, because CL is a multi-factors disease, gene-gene and gene-environment interactions should be considered and brought in. It is possible that specific environmental and lifestyle factors influence the associations between IL-10 and IL-6 polymorphism and CL, including age, sex, diet, smoking, familial history, parasite history, virus and immune factors. Third, whether the CL patients within other complications, such as abnormal liver function, HCC and hepatitis, all the included factors have not been reported. Further comprehensive studies should include above items. Fourth, the stage of CL is not distinguished, which should be analyzed separately (compensatory and decompensated period) and can be more accurate for prediction and treatment.

## Conclusions

Our present meta-analysis suggests that IL-10 -592 and − 1082 polymorphisms may be associated with CL risk, which may be proofed in following larger and comprehensive studies.

## Abbreviations

CL: cirrhosis of liver
LC: Liver cirrhosis
HWE: Hardy–Weinberg equilibrium
OR: Odds ratio
95%CI: 95% Confidence interval
PBC: primary biliary cirrhosis
ALC: alcoholic liver cirrhosis
